# A systems biology approach to investigate the response of *Synechocystis *sp. PCC6803 to a high salt environment

**DOI:** 10.1186/1746-1448-5-8

**Published:** 2009-09-07

**Authors:** Jagroop Pandhal, Josselin Noirel, Phillip C Wright, Catherine A Biggs

**Affiliations:** 1 ChELSI Institute, Department of Chemical and Process Engineering, The University of Sheffield, Mappin Street, Sheffield S1 3JD, UK

## Abstract

**Background:**

Salt overloading during agricultural processes is causing a decrease in crop productivity due to saline sensitivity. Salt tolerant cyanobacteria share many cellular characteristics with higher plants and therefore make ideal model systems for studying salinity stress. Here, the response of fully adapted *Synechocystis *sp. PCC6803 cells to the addition of 6% w/v NaCl was investigated using proteomics combined with targeted analysis of transcripts.

**Results:**

Isobaric mass tagging of peptides led to accurate relative quantitation and identification of 378 proteins, and approximately 40% of these were differentially expressed after incubation in BG-11 media supplemented with 6% salt for 9 days. Protein abundance changes were related to essential cellular functional alterations. Differentially expressed proteins involved in metabolic responses were also analysed using the probabilitistic tool Mixed Model on Graphs (MMG), where the role of energy conversion through glycolysis and reducing power through pentose phosphate pathway were highlighted. Temporal RT-qPCR experiments were also run to investigate protein expression changes at the transcript level, for 14 non-metabolic proteins. In 9 out of 14 cases the mRNA changes were in accordance with the proteins.

**Conclusion:**

*Synechocystis *sp. PCC6803 has the ability to regulate essential metabolic processes to enable survival in high salt environments. This adaptation strategy is assisted by further regulation of proteins involved in non-metabolic cellular processes, supported by transcriptional and post-transcriptional control. This study demonstrates the effectiveness of using a systems biology approach in answering environmental, and in particular, salt adaptation questions in *Synechocystis *sp. PCC6803

## Background

Processes associated with modern agriculture can lead to the loss of irrigated land for crop plant cultivation, due to increasing salinity levels [1]. There has therefore been diverse studies aimed at understanding the susceptibility of higher plants to these adverse conditions, and how several species have developed salt tolerance [2-6]. However, working directly with plants can be disadvantageous due to high cost, long life cycle generation times and limited control over genetic manipulations. Due to their ability to perform oxygenic photosynthesis, cyanobacteria provide favourable models for understanding metabolic processes in higher plants. The unicellular cyanobacterium *Synechocystis s*p. PCC6803 (henceforth referred to as *Synechocystis *unless otherwise stated) has the remarkable capability of surviving in non-saline environments and high saline conditions (up to 1.2 M or ~7% NaCl) [7], and is often the representative species for cyanobacterial salt tolerance studies [8-15]. Hence, *Synechocystis *is a model cyanobacterial candidate for investigating salt adaptation, which could be exploited to provide further understanding of salt adaptation in higher plants.

Deciphering the mechanisms that *Synechocystis *cells adopt to enable adaptation to saline conditions, requires diverse tools in order to gain a systems level understanding. The main body of literature in this area is composed of incubating cells in a saline solution (mainly ~2 to ~4.1% NaCl) and interpreting the physiological changes by following the stress responses on the phenotypic, metabolic, genomic and proteomic level [8-14,16].

DNA microarrays have made valuable contributions in studying the salt responses in *Synechocystis *[10,17]. Kanesaki *et al. *[17] used DNA microarray analysis to monitor changes in transcripts in *Synechocystis *in response to 0.5 M (~3%) salt after 30 minutes. Among the salt induced genes were those that encode for ribosomal proteins, genes associated with the D1 protein at the photochemical reaction centre of PSII, as well as essential genes for biological membrane structure [17]. Marin *et al. *[10] also used DNA microarrays to study *Synechocystis *acclimation to 684 mM (~4%) of salt, 15 minutes, 30 minutes, 2 hours, 6 hours, 24 hours and 5 days after salt addition. This enabled a genome wide profiling of initial salt shock response and longer-term adaptation to salt. Overall, 18.2% of the 3079 genes on the microarray were differentially expressed [10]. Amongst the salt (induced) acclimation genes were those that encode for enzymes involved in glucosylglycerol synthesis and ABC-transporters, both essential for compatible solute accumulation. Interestingly, the temporal response was particularly useful, for example genes encoding for proteins in basic carbohydrate metabolism, were upregulated after 1 day but not after 5 days. Overall, only 39 genes remained differentially expressed after 5 days acclimation [10].

Proteomics is a particularly powerful tool examining adaptation to environmental conditions because proteins are synthesised to allow cells to successfully function in the new environment. A proteomic study on *Synechocystis *approached the subject of adaptation to salt, rather than immediate (shock) responses, by measuring differential expression after 5 days of incubation in ~4.1% NaCl [18]. Using traditional 2DE, 55 salt induced proteins were identified in this cyanobacterium, assessing salt shock response after 2 hours, as well as adaptation after 5 days [18]. Induced proteins included general stress proteins, such as heat-shock proteins. Up-regulation of proteins comprising those from basic carbon metabolism were identified as essential for salt acclimation [18].

A further proteomic study using high throughput quantitative proteomics, rather than traditional 2DE was performed on *Synechocystis *cells adapted to 3% and 6% NaCl in comparison to 0% NaCl [19]. Using the *in vitro *isobaric tags for relative and absolute quantification (iTRAQ) method, potential detrimental effects on major proteomic techniques caused by high salt, e.g. isoelectric focusing and MS, were overcome, and it was found that *Synechocystis *expressed a number of stress related chaperones such as DnaK, GroEL and 60 kDa chaperonin 2 [19].

Recently, it has become popular to study genome and proteome responses to environmental perturbations on a global scale [10,17,18]. It has been shown previously that immediate changes at the gene and protein level, in response to external stresses, can be significantly different than the changes which exist in the adaptive state, and importantly, changes may even be less pronounced [10,18]. A recent study comparing transcriptomic and proteomic data from salt shocked microbial cells also revealed that ca. 40% of the proteomic changes were not detected previously at the RNA level, highlighting the importance in conducting protein-level analyses [18]. It is hypothesised therefore that an increase in knowledge of the salt response in a well-studied system can be generated, using a combined large-scale directed, proteomic and transcript-level study.

This paper therefore focuses on a large-scale survey of the longer-term (9 days and >1 doubling time) response of *Synechocystis *cells to the addition of high (6%) salt using high throughput quantitative proteomics (iTRAQ) in combination with RT-qPCR. This will allow further understanding of the relationship between shock and adaptation, and transcript and protein levels, particularly where post-transcriptional control can be postulated. Discussions of salt adaptive responses are conducted with reference to existing global transcriptomic and proteomic studies.

## Results and Discussion

Following an overview of protein data analysis, the biological results are presented in two main sections. First, the responses that can be mapped as metabolic changes are discussed using the probability-based analysis tool MMG as a guide. Second, significant protein changes involved in non-metabolic cellular responses are interpreted and these form the basis in choosing transcripts for temporal quantitation and hence further interrogation of the salt response in this model organism.

### General Protein data analysis

*Synechocystis *cells were successfully cultured in both conditions of no added salt (0% w/v) and high salt (6% w/v) with exponential growth rates of 0.26 ± 0.01 and 0.11 ± 0.01 d^-1 ^respectively. After addition of 6% salt, mid-exponential growth phase occurred at 9 days, and therefore ensured cells were fully adapted to the new conditions.

In workflow I (Table 1), 77% (24,374) of the total spectra were successfully matched to peptides, and in order to produce accurate protein quantitations, only peptides that were referenced to spectra with = 70% confidence intervals were used. Using a 95% protein confidence cut-off, 207 unique proteins were identified by 6563 distinct peptides.

**Table 1:** A summary of the two different workflows implemented in the proteomic analysis

Analysis stage	Workflow 1	Workflow 2
HPLC	Nano-LC (Dionex)	1200 Series-Chip (Agilent)
Mass spectrometry	ESI-qQ-TOF-MS/MS (AB)	Agilent 6510
Analysis software	Analyst/Bioanalyst^® ^and ProteinPilot™ Software v 2.0 (AB)	MassHunter and Spectrum Mill Software v A.03.03 (Agilent)

Biological and technical variations were calculated and expressed as CV. First, ratios of 115:114 (6% salt compared to 0% salt) and 116:114 (6% salt biological replicate compared to 0% salt) were compared using log_10 _ratios as described previously [20,21]. The mean average CV across all 207 proteins was 0.20 (± 20%), similar to a previous iTRAQ study where 3 data sets across two iTRAQ experiments gave an average biological variation of ± 0.25 [21]. Second, it is expected that the ratio of biological replicate samples 115 (6% salt) and 116 (6% salt) would be close to 1. Using the error factor generated using the ProteinPilot™ algorithm as a weight, the weighted average log_10 _ratio of 116 compared to 115 gave an average ratio of 0.98 (i.e. biological variation is low).

To gain a perspective on the rate of false positive measurement, files were run against a reversed decoy database. In total, 10,722 spectra were assigned to valid peptides (≥ 80% confidence interval) in the correct database search and 24 spectra were identified using the decoy database. Using the method described previously by Elias *et al*. [22] the false positive rate, and hence percentage of peptide-to-MS/MS spectra matches that are possibly incorrect, was measured at 0.4%.

Using Workflow 2 (Table 1), 339 proteins were identified. Spectrum Mill implements a pre-processing step that selects only good quality spectra and merges identical spectral information. Auto-validation of results using an in-built algorithm means only peptides that exceed a set score threshold and percentage peak intensity threshold are used to calculate protein quantitations. Ratios of 115:114 and 116:114 were compared using log_10 _ratios and the mean average CV across all 339 proteins was found to be higher at 0.32 (± 32%) compared with 0.2 for Workflow 1.

Distinct proteins were selected in order to generate a final list of proteins that would be used to interpret biological changes in *Synechocystis *cells in response to salt. Protein identifications and quantitations were only accepted after having manually scrutinised peptide quantitations (two or more MS/MS spectra), mass spectra intensity and quality, as well as examination of biological replicates (as suggested in the General Guideline for Proteomics Data Publication http://www.mcponline.org/) and previous work [19,23]. This produced a final list of 378 proteins for further interpretation, through data sets from both workflows, which equates to 11.9% of total predicted protein coding genes. 165 proteins were identified using both methods, but the majority were unique to workflow 2 (174 proteins). A Pearson correlation coefficient of 0.74 showed good correlation between the 155 proteins that were both identified and quantified in both workflows (using labels 114 and 115 in this instance) (see Additional File 1: Figure S1). The full iTRAQ results and this final list are presented in the [Additional File 2: Table S1].

### Differential Protein Analysis

Additional file 3: Table S2 summarises the differentially expressed proteins identified in this study, and also includes references to previous studies where proteins or corresponding transcripts have been identified as salt regulated in *Synechocystis*. Differential regulation amongst both biological replicates in the iTRAQ experiment was a pre-requisite for further analyses (and therefore two ratios are quoted in the discussion). CV and RV calculations together with previous iTRAQ studies [21,24], were all considered for appointing a suitably stringent significance threshold of 50% fold change. Based on this criteria, 34.7% (131/378) of the identified proteins changed significantly in abundance (between 0 and 6% w/v salt), and hence play potentially noteworthy roles in adaptation to high salinity.

For the following discussion, care was taken to interpret references to previous studies where proteins or corresponding transcripts were previously identified as salt regulated in *Synechocystis*, because of the potential differences in growth and test conditions (for example, Marin *et al *and Kanesaki *et al*. [10,17]). In some cases, not only do the techniques and equipment employed differ (which all contribute to variation) but so does the salt concentrations investigated, the significance thresholds set for differential protein expressions, and the time allowed for cells to adapt to adverse conditions all vary.

### Part A: Metabolic responses

#### Carbon and energy metabolism

Two proteins of carbon metabolism pathways, previously identified as induced in 4% salt, were also induced here, fructose-bisphosphate aldolase (1.52 and 1.55-fold; with probabilities of being up-regulated under high salt conditions (p_+_) of 0.940, 0.965) and phosphoglycerate kinase (up to 3-fold; *p*_+ _= 1.00, 1.00). Dihydrolipoamide acetyltransferase which is part of the pyruvate dehydrogenase complex (post-glycolysis) was also >2-fold up-regulated in high salt (*p*_+ _= 0.624, 1). These changes imply increased requirement for energy as carbon is metabolised, as has been reported formerly [18,25,26]. As expected, a large increase (4.86 and 6.21-fold) in a compatible solute synthesis enzyme, glucosylglycerol (GG) phosphate synthase was observed, an essential response in salt acclimation. An increase in 4-alpha-glucanotransferase (2.78 and 3-fold; *p*_+ _= 0.00, 1.00) has also been seen previously in the long-term [18], and may play a role in making carbon available for GG synthesis. This may also explain the 1.62 and 1.66-fold increase in glucose-1-phosphate adenylyltransferase (*p*_+ _= 0.994, 0.994). These latter two enzymes are positioned close together in pathways involving the re-arrangement of carbon atoms in glycogen synthesis pathways, and the accumulation of glycogen in salt acclimated cells has been reported previously in *Synechocystis *[16].

The cellular response to salt stress implies an increase in energy conversion, however, it is important to note that not all enzymes involved in energy synthesis are necessarily directly related to energy supply in salt stress. A good example of this was reported by Suzuki *et al. *[27], where the kinetic activities of enzymes in the glycolytic pathway were investigated in the mangrove tree in response to long-term 150 mM salt stress. Fructose-6-phosphate transferase (*p*_- _= 0.998, 0.00) was thought to play an important role in the logarithmic growth stage where biosynthesis is rapid, however, it is not thought to be directly related to energy supply for the removal of salt from the cytoplasm [27]. The role of the PPP regulatory control protein OxPPCycle gene appears reduced here (1.52 and 1.69-fold). In support of overall reduced PPP requirement, the reduced expression (2.56 and 2.23-fold) of an enzyme responsible for the hydrolysis of pyrophosphate (PPi), which is formed principally as the product of the many biosynthetic reactions that utilise ATP, suggests an overall reduction in biosynthesis, and linking in with the findings above, these are most likely biosynthesis reactions associated with slowed growth rate in 6% salt.

#### Photosynthesis and respiration

Fifteen proteins, involved in photosynthesis and respiration, were differentially regulated, 13 of which decreased in expression with an increase in salt. As expected, eight are pigment proteins and therefore 6% salt leads to pigment loss in cells, as does 4% salt [18]. In addition, long-term acclimation does not lead to recovery of these pigments. Cytochrome c550 or PsbV decreased 1.93 and 2.13-fold and is associated with the oxygen evolving complex in PSII. Reductions in oxygen evolving machinery have been seen previously at the transcript level in both *Synechocystis *cells (4% salt at 24 hrs) and in *in vitro *isolated thylakoid membranes from *Synechococcus *sp. PCC7942 cells [10,28].

Perhaps the most surprising of the proteins identified with reduced expression was the electron carrier flavodoxin. This protein has previously been associated as an important alternative electron carrier implemented in salt adapted cells of *Synechocystis *(2 to 4% salt) [13,18]. The marked decrease of this protein here implies this role is no longer applicable in higher salt (6%). Its non-essential role has been demonstrated however, when a flavodoxin mutant displayed reduced cyclic electron flow around PSI, but was still capable of acclimation to 2% salt [13].

In PSII, one subunit, CP43 increased 1.82 and 1.80-fold in expression. The increase in PSII reaction protein was unexpected, as previous studies have shown PSII activity to decrease in salt shocked and adapted cells [18,13] (although this particular protein has not previously been identified). It is possible that an initial decrease in levels of this protein may function as damage limitation during initial salt stress, and an increase in expression functions to replace damaged reaction centres when cells have adapted. However, oxidative stress is known to actually inhibit repair of salt-damaged PSII protein D1 in cyanobacteria [29]. These results imply variable stoichiometry in the complexes.

Vipp1 is a protein previously identified as salt induced in *Synechocystis*, and is believed to transfer reaction centre proteins to the thylakoid membranes during stress, and therefore replacement of damaged subunits occurs [30]. Increased expression of replacement proteins has been observed in light stressed cells previously [31]. Finally, apocytochrome f precursor, part of the cytochrome b_6_/f complex, which is involved in electron transfer between both photosystems, increased 1.93 and 1.69-fold in 6% salt. This implies increased electron transfer to PSI (non-cyclic photophosphorylation) and therefore enhanced energy synthesis.

NADH dehydrogenase subunit I is part of the larger enzyme complex, and involved in oxidative phosphorylation in respiration. Despite its 1.59 and 1.75-fold decrease in expression in high salt, other NADH dehydrogenase subunits were identified and not differentially expressed, for example, beta subunit (1.01 and 0.71-fold change), suggesting a stoichiometric change in this complex. A DNA microarray study found transcript levels for subunits of this complex to increase immediately in response to 0.02 M (~1.2%) salt, but decrease in response to 1.0 M (6%) salt.

#### MMG analysis

MMG analysis was used to build a systems picture of the metabolic response to salt stress. The proteomic data was mapped onto the KEGG metabolic network (accessed 13^th ^August 2008) using a Perl script and the probabilities were computed using the R module 'MMG' (http://cran.r-project.org/, R v2.8.1, MMG v1.4). The parameter σ was set to 0.2, by fitting the central part of distribution of the 115:116 log_10 _ratios to a Gaussian distribution. The parameter α was set to 1, as advised in the original paper [32]. MMG helps to interpret the proteomic data in light of the metabolic network's topology. This allows the identification of regulated enzymes, even when they have not been quantified or when they were quantified and did not exhibit a marked abundance change, which still could be biologically significant.

MMG's 1/λ statistics give insights into the degree of regulation: for down-regulated proteins, 1/λ = 0.73 (SD 0.10), while, for the up-regulated proteins, 1/λ = 0.61 (SD 0.09). This indicates that more down regulated processes can be detected by MMG.

**Figure 1:**
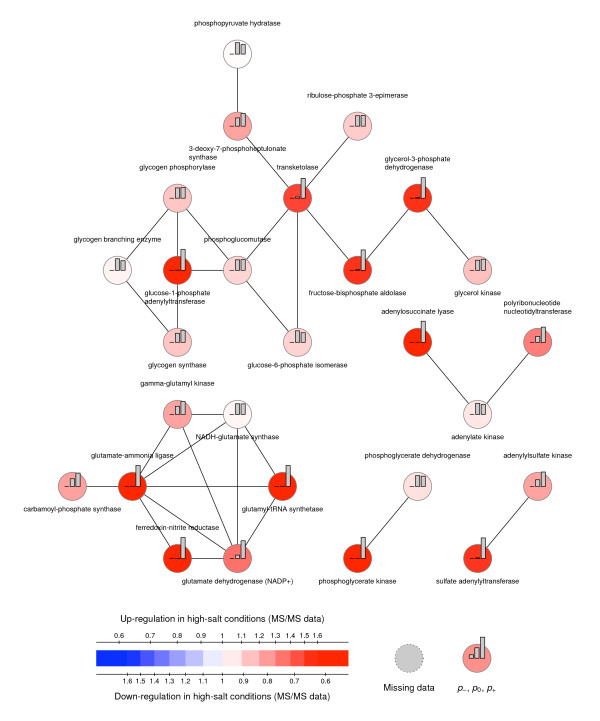
**Protein networks up regulated under high salt conditions identified using Mixed Model on Graphs (MMG)**. The nodes represent the single-unit and multiple-unit enzymes in *Synechocystis*'s metabolic network, as per the KEGG database. Two enzymes are connected when a product of the reaction catalysed by one enzyme is a reactant of the reaction catalysed by the other enzyme.

**Figure 2:**
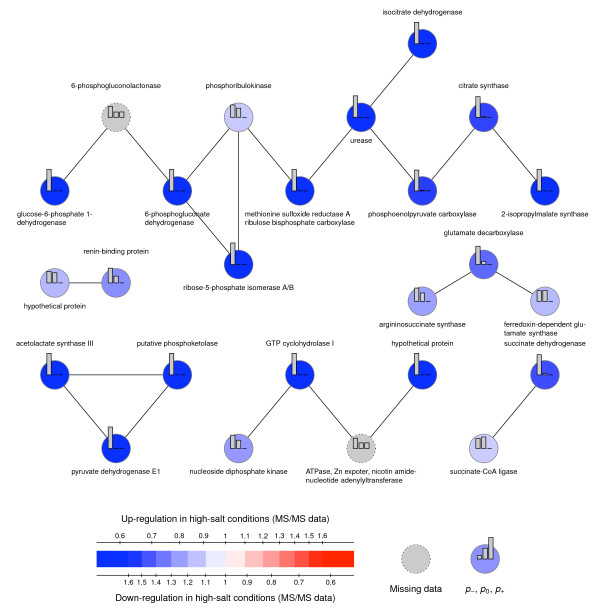
**Protein networks down regulated under high salt conditions identified using Mixed Model on Graphs (MMG)**. The nodes represent the single-unit and multiple-unit enzymes in *Synechocystis*'s metabolic network, as per the KEGG database. Two enzymes are connected when a product of the reaction catalysed by one enzyme is a reactant of the reaction catalysed by the other enzyme.

The networks identified by MMG using the dataset resulting from Workflow 2 are presented in Figure 1 (up regulated in high salt conditions) and Figure 2 (down regulated in high salt conditions). They were obtained by selecting proteins with a probability of being up-regulated, *p*_+_, greater than 0.45 (for the up-regulated network) and a probability of being down-regulated, *p*_-_, greater than 0.45 (for the down-regulated network). The nodes in Figure 1 and 2, represent the single-unit and multiple-unit enzymes in *Synechocystis' *metabolic network, as per the KEGG database. Two enzymes are connected when a product of the reaction catalysed by one enzyme, is a reactant of the reaction catalysed by the other enzyme. Edges are weighted during the MMG analysis, and the weights are inversely proportional to the frequency of the compound within the metabolic network in order to give less prominence to currency metabolites such as ATP, ADP, NADPH, etc.

Among the proteins found to be down-regulated by MMG, the 6-phosphogluconolactonase (*p*_- _= 0.5) was found. It complements the pathway made of quantified enzymes (namely, glucose-6-phosphate 1-dehydrogenase, 6-phosphogluconate dehydrogenase, and ribose-5-phosphate isomerase A/B) from the oxidative phase of the pentose phosphate pathway, whereby β-D-glucose-6-phosphate is turned into D-ribose-5-phosphate. The non-oxidative phase, on the other hand, of the pentose phosphate pathway is found to be up-regulated. The reasons for this discord in both stages in the PPP require further investigation, but the requirement of increased carbon fixation may be an important factor.

Among the proteins found to be up-regulated by MMG, two enzymes are identified that are related to glycogen synthesis and degradation: the glycogen synthase, the glycogen phosphorylase, and the glycogen branching enzyme. Since the glycogen synthase and the glycogen phosphorylase function in opposition, the combined effect of their up-regulation is unclear. However, the relationship between carbon requirement for glucosylglycerol synthesis and carbon excess being converted to a carbon store in the form of glycogen, is likely to be determined by adaptation time, where the need for compatible solute diminishes.

**Figure 3:**
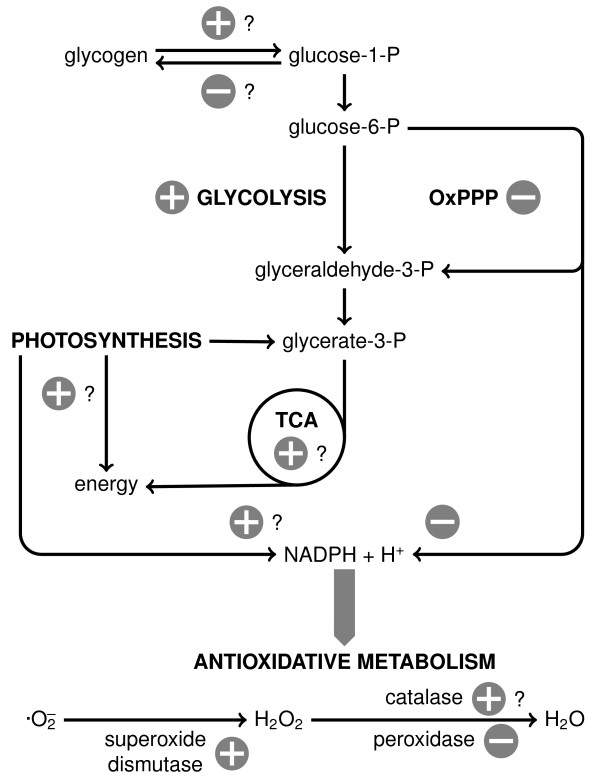
**Metabolic changes in salt adapted cells**. A summary of metabolic changes occurring in *Synechocystis *cells adapted to 6% salt using iTRAQ-based global proteome analysis.

Due to the connection between high salt response and oxidative stress, this study prompts the question of the existence of producing and consuming pathways of NAD(P)H. Whereas superoxide dismutase is manifestly up-regulated in both Workflows (2.83 and 2.83 in Workflow 1, 2.44 and 2.36 in Workflow 2), further detoxification of H_2_O_2 _is not clearly up-regulated. Peroxidase is indeed found to be down-regulated (0.58 and 0.50 in Workflow 2), whereas the catalase is only mildly up-regulated (1.06 and 1.21 in Workflow 1, 1.45 and 1.39 in Workflow 2). Down-regulation of the oxidative phase of the pentose phosphate pathway could mean the depletion of reducing power, which could be compensated by photosynthesis. Figure 3 summarises the metabolic changes that occur in cells when adapted to 6% salt.

### Part B: Non-metabolic responses

#### Stress proteins

In this study, relative quantitations show that several of these proteins are required in elevated amounts in *Synechocystis *cells adapted (for exactly 9 days) to 6% salinity. A 2.24 and 1.90-fold induction was seen in heat shock protein 90 (Hsp90), and this particular stress protein has not been identified as salt induced in previous proteomics experiments in the short or long-term [19]. Hsp90 contributes to an array of cellular processes, including protein degradation, protein folding and even signal transduction [19,33]. Its gene transcript levels have been seen to increase in response to ~3% salt [17] and 4% salt [10] in the short-term. In the latter study by Marin *et al. *[10], transcript levels were not induced after 5 days.

Anti-oxidative enzyme superoxide dismutase was up-regulated over 2-fold in all experiments (Additional file 3: Table S2), and this response has also been seen in the short-term and after 5 days at the protein level [18]. A similar scenario was seen for molecular chaperone DnaK, although fold increases were less than for superoxide dismutase. Although the induction in DnaK in response to high salt (4% and 6%) has been seen previously at the protein level [18,19], elevated transcript levels have not been observed in the long-term [10]. It is not possible to extrapolate whether this gene is transcribed in the long-term, and therefore *dnaK *was a candidate for the RT-qPCR analysis as discussed below. The stress chaperones, 16.6 kDa small heat shock protein and GroES (co-chaperonin) were both expressed higher in 6% salt (1.75/2.10-fold and 1.59/1.83-fold, respectively), however, previous studies have identified transcript and protein levels to be salt responsive in the short-term only [10,18]. Not all stress proteins were expressed in higher amounts in 6% salt and heat shock protein, GrpE, was reduced by 1.66 and 1.72-fold.

#### Transport and binding proteins

In contrast to the results observed in *Synechocystis *cells adapted to 4% salt (over 6 to 8 days), four nutrient binding proteins experienced large reductions in expression in 6% salt. Relative levels of iron transport protein (1.75 and 1.99-fold), periplasmic phosphate binding protein (10.07 and 10.38-fold), periplasmic iron binding protein (9.28 and 11.08-fold) and a bicarbonate transporter (2.09 and 3.62-fold) were all significantly diminished. Moreover, bacterioferritin, which is responsible for storage of as much as 50% of cellular iron, was 1.61 and 1.72-fold reduced. It is not certain why cells reduce expression of transporter proteins in high salt (6%). Reduced growth and overall protein synthesis may mean fewer nutrients are required, although further work needs to be done to elucidate the specific reasons for this unexpected response.

Three transport proteins did increase in expression in 6% salt. An ABC-1 like transporter increased 4.27 and 4.93-fold, and is considered to be a putative ubiquinone biosynthesis protein and therefore could be implemented in energy generation. This protein, or its coding gene, has not previously been identified as differentially regulated during salt stress. Another ABC transporter also increased 2.35 and 2.66-fold. Finally, a 1.83 and 1.52-fold increase in nitrate transport protein was observed. Interestingly, the uptake of combined nitrogen has previously been suggested to inhibit Na^+ ^ion influx, and therefore a mechanism for the protection against salt stress in the freshwater and brackish water cyanobacteria, *Anabaena *sp. strain L-31 and *Anabaena torulosa *[34]. This response however, has not previously been reported in *Synechocystis*.

#### Transcription and Translation

Overall, 26 proteins involved in transcription and translation were identified as differentially expressed in this study, and 13 of these proteins experienced over a 2-fold change (in at least one replicate).

A 2.91 and 3.17-fold increase in elongation factor EF-G may occur for synthesis of so called 'stress proteins' required for cell survival. Cellular stresses like salt stress which lead to global repression of translation, are often accompanied by increased translation of selected proteins that are required for cell survival. It is possible that increased levels of mRNA coding for stress-proteins, compete more successfully for protein synthesis ability in salt stressed cells, than transiently expressed mRNA.

Proteins were differentially expressed in this long-term adaptation study which had only previously been shown to change in the short-term (at transcript and protein level [10,18]), for example, a 2.21 and 2.22-fold increase in RNA-binding protein. It is possible that this protein assists in stabilising nucleic acid structures that are susceptible to damage caused by changes in water potential and ionic strength. Another protein that assists in tackling the detrimental effects of salt in the long-term is peptidyl-prolyl cis-trans isomerise (2.07 and 2.40-fold increase). This enzyme accelerates protein folding [35]. Finally, the 1.83 and 2.32-fold induction of glutamyl-tRNA synthetase (p_+ _= 1, 0.99394) has been observed previously in the long-term [18].

#### Other proteins of interest

This shotgun study identified several proteins that could collectively play an important role to assist a cell's survival in high salt. Cell envelope enzyme UDP-N-acetylmuramoylalanyl-D-glutamyl-2,6-diamino-pimelate-D-alanyl-D-alanine ligase (p_+ _= NA, 0.9918) is thought to regulate cell shape by controlling the cell wall structure [36]. The cell wall in *Synechocystis *cells and all bacteria protects the cell from the environment by providing rigidity to counteract internal osmotic pressure. This enzyme is specifically involved in peptidoglycan synthesis, and it is this that provides strength. It is probable that the change in external salt concentration means this enzyme is required in larger amounts to produce more peptidoglycan and help maintain the overall shape of the cell, and confidence in this possibility is increased because of the 2.57 and 2.72-fold increase in enzyme glutamate--ammonia ligase (p_+ _= NA, 0.9965), which functions in the same pathway. Both proteins have not previously been identified in the salt response, however, genes in the same pathway have been shown to be induced in heat shocked *Synechocystis *cells [37], and an increase in peptidoglycan layers for protection was supposed.

Glycerol-3-phosphate dehydrogenase was also identified as salt induced (1.95 and 1.52-fold; p_+ _= 0.998, 0.97375) for the first time using proteomics, and is thought to play a role in compatible solute accumulation. This enzyme coding gene, has been induced in the short-term and long-term [10], and its transcript levels were also investigated.

Anti-sigma B factor antagonist was 2.70 and 2.30-fold less expressed in high salt, and despite not being identified previously at the protein or transcript level, this change can be expected. This protein is involved in terminating transcription of the general stress regulon and therefore is not required in 'stressed' conditions. Its mode of action has been studied in *Bacillus subtilis*, and it is assumed that a reduced amount of anti-sigma B factor makes the stress response process more efficient in adapted cells.

#### Gene expression analysis

Currently, understanding of the *Synechocystis *systems response to salt cannot be achieved using MMG for proteins that are not mapped to metabolic pathways. Therefore, an extra level of investigation was included for a selection of differentially expressed non-metabolic proteins by performing qRT-PCR on their respective transcripts. Many proteins acknowledged to play a role in salt acclimation have been discovered previously in the long-term, short-term and at both the protein and transcript level, for example, superoxide dismutase (Additional file 3: Table S2. However, there were also non-metabolic proteins identified in this study, which had opposite fold changes to previous studies. A selection of non metabolic protein coding genes were therefore analysed at the transcript level based on the following premises

i) Proteins not previously associated with salt response (novel study).

ii) Proteins that were controlled in the opposite direction to previous studies (contradictory study).

iii) Proteins or genes previously associated with salt response (confirmatory study).

iv) Proteins or genes with differences between short-term and long-term responses (shock vs. adaptation study)

**Figure 4:**
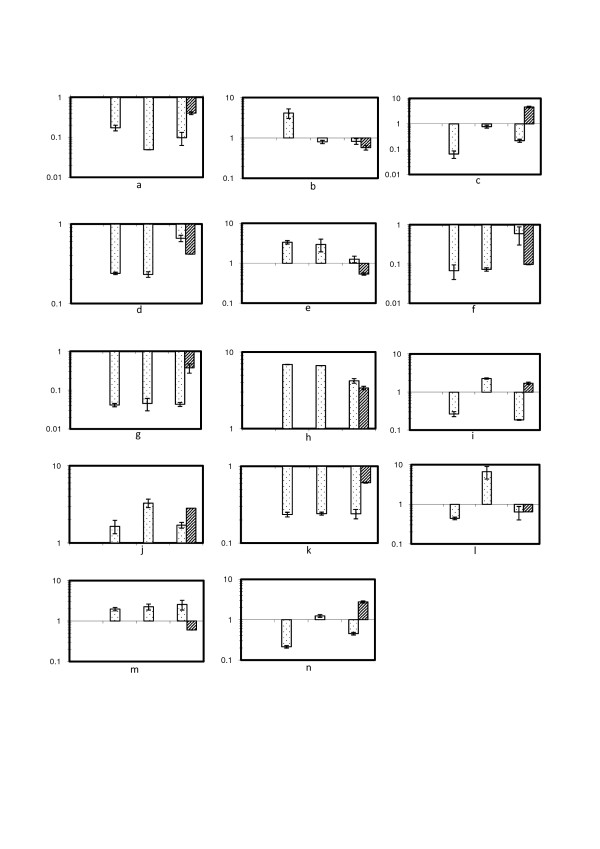
**Gene expression changes for non-metabolic protein encoding genes**. The dotted bars correspond to RT-qPCR results and the dashed bars signify iTRAQ results. In each plot the x-axis denotes sampling time points at t = 0 hrs, 2 hrs, 24 hrs and 9 days. Inductions are relative to t = 0 (0% salt). Error bars represent standard deviations (n = 2). (a): Anti-sigma factor B antagonist; (b): Ribosome releasing factor; (c): ABC-1 like protein; (d): ATP-dependent Clp protease; (e): Glutathione peroxidise; (f): Periplasmic phosphate binding protein; (g): Bicarbonate transporter; (h): Circadian clock protein; (i): DnaK; (j): Superoxide dismutase; (k): SOS regulatory protein; (l): Hypothetical protein sll1106; (m): Cell division protein; (n): Co-chaperonin GroES.

In total, 14 protein-coding genes were quantified across the time-series and similar to the iTRAQ data, were only treated as significant if both biological replicates exceeded the 1.5-fold threshold for extra stringency. Figures 4a-n show the gene expression changes (dotted bars) after incubation in 6% salt, after 2 hours, 24 hours and 9 days. The dashed bars signify iTRAQ results after only 9 days. The values are all relative to time = 0 or 0% salt, and therefore the first value is recorded as 1 in all cases.

#### Novel protein level changes

RT-qPCR was performed on four protein-coding genes that have not previously been associated with a salt response (Figure 4a-d). Anti-sigma B factor antagonist has not previously been identified as an essential salt response protein, and Figure 4a illustrates how the gene transcript levels (*icfG*) decrease after only 2 hours in 6% salt, and remains approximately at this level until cells are fully acclimated. This is the first time to our knowledge that this protein has been identified in a proteomics salt stress study in *Synechocystis*, so it is not possible to ascertain whether its reduced expression is related to the *extent *of salt stress.

Figure 4b shows the regulation of the gene encoding the ribosome releasing factor and the corresponding protein. The results between the differential expression based on gene and protein level on day 9 correlate well with reduced expression. An immediate increase in transcript (*frr*) level occurs after 2 hours of salt stress. This may be due to cells initiating the transcription and translation of general and salt stress specific proteins crucial for long-term adaptation.

The first example of poor correlation between transcript and protein quantitation occurs with an ABC-1 like protein (UbiB) (Figure 4c). It was induced 4.27 and 4.93-fold in high salt and therefore postulated to play a significant role in salt acclimation. Transcript data shows an immediate reduction in expression (2 hours). After a recovery to approximately initial levels (24 hours), the expression of the gene after 9 days is again reduced and this final quantitation contrasts vastly with protein levels.

ATP-dependent Clp protease proteolytic subunit is involved in degrading protein aggregates during stress and can act as a molecular chaperone [38]. It would therefore be expected to increase in expression in stressed cells, however, in 6% salt it decreased 2.38 and 2.39-fold. Figure 4d shows that transcripts levels for this gene (*clpP*) also decrease immediately in salt-stressed cells, and remain reduced after 9 days, supporting the assumption that this subunit does not play a role in acclimation to 6% salt.

#### Protein level changes in contrast to previous studies

RT-qPCR was performed on three protein-coding genes that were controlled in the opposite direction to previous studies (Figure 4e-g).

Glutathione peroxidase is an enzyme whose principal function is to protect cells against damage caused by endogenously-formed hydroxyperoxides (p_- _= NA, 0.99985). This type of cell damage is often a result of secondary stress associated with ROS [39] A 3-fold increase in this protein was observed previously in 4% salt stressed cells [18], as well as a transient increase in transcript levels [10]. In agreement with these previous studies, the RT-qPCR results also showed a transient increase in transcript (*gshP*) levels (Figure 4e). It seems likely that after the initial response to oxidative stress, cells that are acclimated no longer require this stress enzyme and therefore there is a decrease in abundance in this iTRAQ study.

A very large decrease in periplasmic phosphate binding protein was observed in the iTRAQ study (10.07 and 10.38-fold). An ABC-transporter which binds phosphate was identified as newly induced in cells adapted to 4% salt [30], and therefore implies that the requirement for phosphate changes significantly in cells adapted to higher salt concentrations (6% salt). Figure 4f shows that transcript levels (*pstS*) for this protein are only transiently decreased, and in contrast to protein levels, it recovers when approaching adaptation (9 days). This effect of higher salt concentrations on transport proteins is demonstrated further where bicarbonate transporter protein is reduced 2.09 and 3.62-fold in 6% salt. Transcript (*cmpA*) levels are also stably reduced according to RT-qPCR results (Figure 4g). As with phosphate binding protein discussed above, previous studies show the protein to be newly induced in 4% salt [30], and transcript levels to be over 5-fold induced after 6 hours [10].

#### Confirmatory protein level changes

RT-qPCR was performed on four protein coding genes which have previously associated with salt response (Figure 4h-k).

The gene encoding for the circadian clock protein (*kaiC*) is known to increase in 4% salt in the long-term (5 days) [10], but has not been quantified at the protein level formerly. iTRAQ results show a 3.23 and 3.58-fold increase after 9 days, but in contrast to the study by Marin *et al. *[10], transcript levels quantified here were induced in the short-term too (Figure 4h). Fold changes of both transcript and proteins were very similar after 9 days.

Two further gene transcripts, *dnaK *and *sodA*, were quantified for confirmatory purposes, and code for stress chaperone DnaK and anti-oxidative enzyme superoxide dismutase, respectively. DnaK has previously been induced during short and long-term responses to 4% salt [18], and its induction in iTRAQ concurs. Also, the immediate response at transcript level to ~3% salt [17] was seen using RT-qPCR at 24 hours (Figure 4i). However, in this study, its transcript and protein levels were in complete disagreement after 9 days, and this could indicate post-transcriptional control. The same comparison with superoxide dismutase and SOS regulatory protein (LexA protein) produced more complementary results (Figures 3j and 3k).

#### Differences in short-term and long-term changes

RT-qPCR was performed on three protein-coding genes where discrepancies arose in regard to short-term and long-term responses between previous work with microarrays, 2DE gels and iTRAQ data presented here (Figure 4l-n).

Hypothetical protein sll1106 does not currently have a putative function, but control of its gene transcription was shown to be 12.6-fold enhanced in ~3% salt using a DNA microarray in the short-term [17], The corresponding protein was quantified by iTRAQ and found to decrease 1.57 and 1.51-fold after 9 days, and these results pose the question of whether this protein is only important for a short-term response to salt stress. RT-qPCR results seem to suggest this is the case, with an increase in transcript level only seen after 24 hours (Figure 4l). Relative changes in transcript and protein levels were in agreement after 9 days. Cell division protein FtsZ was observed to be 1.72 and 1.62-fold reduced in expression in high salt using iTRAQ, and this was expected as growth rate slows. Despite this, Kanesaki *et al. *[17] reported a short-term increase in this protein in ~3% salt. RT-qPCR and iTRAQ results contrasted completely at 9 days, with transcript levels reduced and protein levels induced, and therefore post-transcriptional control may be involved in cell division (Figure 4m). Cell growth in coordination with cell division is a process thought to be under control of translation regulation [40]. The initiation of translation is a complex process, and is the main target for post-translational control [40]. Finally, protein and transcript levels of stress co-chaperonin GroES have been seen to be induced only in the short-term previously [10,18] whereas iTRAQ results show a 2.61 and 2.93-fold increase after 9 days. Analysis of RT-qPCR data shows a general repression in transcription of the *groES *gene, despite a brief return to 'normal' levels after 24 hours (Figure 4n). Again, the possibility of post-transcriptional control in expression arises to explain its accumulation after 9 days.

## Conclusion

In this study, *in vitro *isobaric labelling was applied to gain an insight into how *Synechocystis *cells adapt to high salt (6%) over an acclimation period of 9 days. Using the quantitative power of iTRAQ, protein abundance changes were related to essential functional alterations in the cell system, which enabled survival in these adverse conditions. Many of these changes have been observed in previous studies using alternative proteomic tools, but novel changes were also discovered. For example, increase in energy demand for active extrusion of salt ions and re-directing control of carbon metabolism enzymes for compatible solute synthesis was observed, but with proteins not quantified previously. Newly identified responses were also attributed to the identification strength of shotgun proteomics workflow, for example, the reduced expression of anti-sigma B factor. Moreover, in the salt concentrations of this study, unexpected results included less reliance on alternative electron flow carrier, flavodoxin, and a reduced requirement for several transport and binding proteins. MMG analysis of metabolic pathways strengthened hypotheses associated with elevated energy demand and the alleviated requirement for reductive power through a down-regulated oxidative PPP cycle.

The two workflows implementing different peptide fractionation systems, mass spectrometers and analysis software, produced complementary results (r = 0.74) and a list of proteins identified with high confidence was collated. Unlike previous proteomic studies assessing the immediate salt response in cyanobacteria, concentrating on adaptation of cells meant smaller expression changes were expected (50%). An average CV of 0.20 and 0.32 across the two workflows, from all proteins sourced from biologically replicate cells, made this possible. Therefore higher quantitation accuracy, often associated with well-designed transcriptome studies, can be achieved with proteomics.

The relative transcript abundance of 14 protein-coding genes presented here were also temporally quantified to further analyse the functional responses postulated using non-metabolic protein abundance changes. The majority of proteins (9/14) and corresponding measurements of mRNA levels were in accordance, and hence could be used in combination to derive insight into how *Synechocystis *cells acclimate to 6% salt. However, there was strong discordance between protein and transcript fold changes for five of the candidate genes, for example, in the molecular chaperone DnaK. Such large variations imply that protein abundance changes cannot be predicted by examining gene expression data alone, and therefore a systems biology approach that combines both proteomics and transcriptomics provides deeper understanding.

## Materials and methods

All chemicals were purchased from Sigma-Aldrich (Gillingham, Dorset, UK) unless otherwise stated.

### Culturing and cell preparation

Replicate flasks of axenic cells of *Synechocystis *were grown in batch culture as described elsewhere [19]. For protein extraction, two biological replicate cultures were grown in BG11 media with no additional salt added, and 50 mls were harvested during mid-exponential phase (in the mid-light cycle) at 10,000 × g for 20 minutes at 4°C. Another set of biological replicate cultures were grown in BG11 media supplemented with 6% (w/v) salt (NaCl), and 50 ml were harvested during mid-exponential phase for protein extraction.

For RNA extraction, biological replicate cultures were grown in BG11 media only as described above. Immediately prior to supplementation with 6% (w/v) salt, 50 ml of cells were harvested (calibrator sample t = 0). 50 ml of cells were also harvested after 2 hours, 24 hours and 9 days after the addition of salt. Pelleted cells for RNA extraction were stored in *RNA*later solution (Applied Biosystems, Framingham, MA, USA) at -80°C.

### Protein extraction and labelling

Following the method outlined by Pandhal *et al. *[19], harvested cells were washed 5 times with sucrose based buffer and resuspended in 1 ml of freshly prepared Tris-based extraction buffer before extracting the proteins via liquid nitrogen freezing with mechanical cracking. The supernatant (soluble proteome) was recovered using centrifugation and quantified as described previously [19]. 100 μg of precipitated protein per phenotype was re-solubilised with 0.5 M triethylammonium bicarbonate (TEAB) buffer at pH 8.5 prior to protein digestion.

### iTRAQ labelling and peptide fractionation

The iTRAQ method, which has successfully been applied to cyanobacterial studies of high salt adaptation, was adopted here for the proteomic analysis, with the protocol described in detail elsewhere [19]. In this case, iTRAQ labels 114, 115 and 116 corresponded to phenotypes grown in low salt (0% w/v NaCl added), high salt (6% w/v NaCl added) and high salt (6% w/v NaCl added) (biological replicate), respectively. After labelling, all three samples were combined and vacuum evaporated prior to strong cation exchange (SCX) HPLC separation. The gradients, buffers and conditions used for SCX fractionation have been described in detail previously [19].

### LC-MS/MS analysis

The SCX fractions were analysed using two separate workflows (see Table 1). Similar to previous studies [23,19], for Workflow 1, each SCX fraction was resuspended in low organic buffer, Buffer I (3% acetonitrile, 0.1% formic acid) and loaded onto a nano-LC (Dionex, LC Packings, The Netherlands). A 102 min gradient using high organic buffer, Buffer II (97% acetonitrile, 0.1% formic acid) eluted samples from a 0.075 × 150 mm analytical column (3 μm C18 Dionex-LC Packings) directly into a QSTAR XL ESI qQ-TOF-MS/MS (Applied Biosystems; MDS-Sciex) at 300 nL/min. The programme consisted of 5 min of 5% Buffer II, increased to 30% over the next 98 min, followed by ramping to 90% Buffer II over 4 min, which was then held for 7 min. Subsequently Buffer II was reduced to 5% for washing and held 12 min. Each MS survey scan was performed at 1 Hz over a 300-200 m/z range. MS/MS scans were performed on two selected precursors over a 65-1600 m/z range. Under the manufacturer's (Applied Biosystems) instructions, an elevated rolling collision energy range was incorporated to achieve optimal fragmentation (increment of 10 eV compared to standard settings). Samples were injected twice to increase proteome coverage and reliability [20].

In workflow 2 (Table 1), the exact same SCX fractions were also run on a different HPLC and MS instrument with different analysis software. The Agilent 1200 Series HPLC-Chip/MS system which combines a trap column with an analytical column (3 μm diameter C18 column, 0.075 × 150 mm) on a chip was used with the same buffers as the nano-LC described above, except trifluoroacetic acid was used in place of formic acid. In addition, the same gradient (buffer %) described above was used but shortened to 30 mins (ca. one third of the time). This system also used nanospray at 300 nL/min for delivery into an Agilent 6510 ESI-Q-TOF MS (Agilent Technologies, Waldbronn, Germany). Each MS survey scan was performed at 1 Hz, but MS/MS scans were performed on three selected precursors, both over the m/z ranges.

### iTRAQ data analysis

In workflow 1, output files from the ESI-qQ-TOF-MS/MS were analysed using ProteinPilot™ Software v 2.0 (Applied Biosystems, MDS Sciex), employing the Paragon Search algorithm [41]. A *Synechocystis *proteome database was used to search all spectra (3264 entries, retrieved from NCBI, March 2007). All spectra were searched against the database using a defined cysteine-fixed modification, mass tolerance of 0.1 Da MS, 0.15 Da MS/MS and undefined mis-cleavage tolerances, so that all possible cleavages would be considered. The generated results included full peptide and protein lists. A cut-off value of 70% was assigned to peptide calculations, and therefore only these would be used to calculate final protein quantitations. Moreover, only proteins with >95% confidence were considered further. Normalisation of data was carried out using the software in-built method of adjusting the median ratio of all quantitations towards unity. This is based on the assumption that most proteins would not change in abundance under perturbed conditions.

For Workflow 2, data acquisition using Agilent 6510 mass spectrometer was performed using Mass Hunter workstation. Spectrum Mill Software version A.03.03 was then used for peptide and protein identification and quantitation using the same settings as above. Normalisation of the median towards unit was performed manually.

To provide a measure of the potential false positive rate for protein identification, a decoy database was used consisting of reversed (*Synechocystis*) proteome sequences [22], created using a Perl script. Two reversed cyanobacteria proteomes were included (*Nostoc punctiforme *PCC73102, 7672 open reading frames (ORFs) and *Nostoc *sp. PCC7120, 6130 ORFs, as well as reversed *Synechocystis*, 3264 ORFs). All were retrieved from NCBI RefSeq, March 2007. An analysis of coefficient of variance (CV) amongst biological replicate samples was used as a guide to select only significantly regulated proteins.

The network modelling approach 'mixture model on graphs' (MMG) [32,42] was also used on the metabolic network of *Synechocystis *as given by KEGG (accessed 27 Jan 2009) with σ = 0.2 (estimated by fitting a Gaussian distribution). Probabilities are given whenever possible (*p*_+ _for proteins up-regulated under high-salt conditions and *p*_- _for proteins down-regulated under high-salt conditions). The geometric mean of the replicates is used for each protein. Two probabilities are given depending on the workflow used to identify and quantify the proteins. These probabilities were used to ascertain the reliability of the quantifications discussed in the text.

### RNA extraction

Total RNA extraction was achieved using a modified version of the RNeasy kit (Qiagen, West Sussex, U.K). In order to generate time series information, 50 ml cells were harvested (centrifugation at 5000 × *g *for 5 min at 4°C) during mid-exponential phase in (i) t = 0 (no salt added), (ii) t = 2 hr (2 hours after 6% salt addition), (iii) t = 24 hr (24 hours after 6% salt addition) and finally (iv) t = 9 days (9 days after 6% salt addition). Biological replicate samples were treated in parallel. These cells were further washed three times with sucrose based buffer for 3 min in a cell disruptor (Scientific Industries, NY, USA) followed by 3 min centrifugation at 19,000 × *g*. The manufacturer's protocol (Qiagen) was followed after the final centrifugation step and the extracted RNA was stored at -80°C.

### DNase treatment, RNA quantitation and cDNA synthesis

Contaminating DNA in RNA samples was removed with application of Turbo DNA-*free *(Ambion, Applied Biosystems, Framingham, USA) as described in the manufacturer's protocol. The integrity and size distribution of the DNA-free RNA extract was checked using agarose gel electrophoresis (1.2% agarose gels made up using 1 × TAE. (Tris-acetate-EDTA) buffer and 0.5 μg ml^-1 ^ethidium bromide). RNA quantitation was performed using RNA-specific fluorescence with the Qubit™ Quant-iT system (Invitrogen, U.K.). Synthesis of cDNA from the total RNA extract was achieved using Quantitect kit (Qiagen).

### Primer design and RT-qPCR

Primers were designed using Primer Express Software version 3.0 (Applied Biosystems) (full sequences and optimal annealing temperatures are given in additional material (Additional File 4: Table S3). Genes of interest were selected for primer designing following protein quantitation (iTRAQ) analysis. Ribosomal gene 23S (*rrn23Sa*) was used as the endogenous control gene, and suitability of this gene (insignificant variance across the test conditions) was checked using the 'absolute quantitation' mode on the 7500 FAST real-time PCR system (Applied Biosystems). This mode was also used to optimise PCR efficiency for all primer pairs to within 90-110% and ensure that no non-specific amplification occurred. The 'relative quantitation' mode was used to calculate the expression changes in genes relative to the calibrator sample (0% salt, t = 0). Power SYBR green (Applied Biosystems) was implemented for RT-qPCR reactions which contains internal ROX dye to normalise any resulting fluorescence in each reaction.

The amplification programme was run with the following steps (i) 95°C for 10 min, (ii) quantification (× 40 cycles) (95°C for 15 s, and 60°C for 1 min). A dissociation step was included to verify the specificity of each reaction by displaying a change in fluorescence over an increase in temperature. All reactions included a technical replicate that enabled standard deviations of quantitations to be calculated, and contamination was assessed by adding no template controls for each gene. Amplification of the endogenous control gene was performed on each plate for extra accuracy and reliability in the quantitations. Data capture was accomplished using FAST system SDS software version 1.4. Relative quantification of gene expression was achieved using the ΔΔC_T _method [43]. Interpretation of the cycle number where fluorescence reaches a particular threshold produced a relative expression of a given gene, which was normalised against the endogenous control.

## Competing interests

The authors declare that they have no competing interests.

## Authors' contributions

JP designed and carried out the proteomic and transcriptomic studies, analysed the data and drafted the manuscript. JN conducted the analysis using MMG and helped draft the manuscript. PCW participated in the design of the study, analysis of the data and drafting of the manuscript. CAB participated in the design and coordination of the study, analysis of the data, and drafting of the manuscript. All authors read and approved the final manuscript.

## Supplementary Material

Additional file 1 **Figure S1**. Correlation of quantitations for the 155 proteins identified and quantified across Workflow 1 and 2 for labels 114 and 115. R = 0.74 (PP-ProteinPilot^®^, SM-SpectrumMill). Click here for file

Additional file 2 **Table S1**. Full iTRAQ results. Click here for file

Additional file 3 **Table S2**. Differentially expressed proteins in *Synechocystis *cells adapted to 6% salt, quantified using isobaric tags. Click here for file

Additional file 4 **Table S3**. Primer (oligonucleotide) sequences designed for each protein-coding gene (T_a _= annealing temperature). Click here for file
