# Supplementary material for: A systems biology approach to investigate the response of Synechocystis sp. PCC6803 to a high salt environment
Source: Saline Syst. 2009 Sep 7;5:8. doi: 10.1186/1746-1448-5-8 (PMC2743698; doi:10.1186/1746-1448-5-8)
Supplement: Additional file 1 — Figure S1. Correlation of quantitations for the 155 proteins identified and quantified across Workflow 1 and 2 for labels 114 and 115. R = 0.74 (PP-ProteinPilot®, SM-SpectrumMill). [file 1746-1448-5-8-S1.doc]

Figure S.1 Correlation of quantitations for the 155 proteins identified and quantified across Workflow 1 and 2 for labels 114 and 115. R = 0.74 (PP- ProteinPilot®, SM- SpectrumMill).
