# Supplementary material for: A systems biology approach to investigate the response of Synechocystis sp. PCC6803 to a high salt environment
Source: Saline Syst. 2009 Sep 7;5:8. doi: 10.1186/1746-1448-5-8 (PMC2743698; doi:10.1186/1746-1448-5-8)
Supplement: Additional file 3 — Table S2. Differentially expressed proteins in Synechocystis cells adapted to 6% salt, quantified using isobaric tags. [file 1746-1448-5-8-S3.doc]

Table S2: Differentially expressed proteins in *Synechocystis* cells adapted to 6% salt, quantified using isobaric tags.

| **gI accession** | **Name** | **Biological rep. 1** | | | **Biological rep. 2** | | | **Functional groups** | **Observed previously at protein level** | | **Observed previously at transcript level** | |
| --- | --- | --- | --- | --- | --- | --- | --- | --- | --- | --- | --- | --- |
|  |  | **Ratio** | | **CV** | **Ratio** | | **CV** |  | **reference/ no** | **short/ long term** | **reference/ no** | **short/ long term** |
| **INCREASED EXPRESSION IN 6% ACCLIMATED CELLS** | | | | | | | | | | | | |
| 16329461 | ferredoxin-sulfite reductase | 1.82 | | 0.24 | 1.57 | 0.24 | | Amino acid biosynthesis | no |  | no |  |
| 16330927 | sulfate adenylyltransferase | 1.55 | | 0.28 | 1.51 | 0.24 | | Amino acid biosynthesis | no |  | no |  |
| 16331104 | glutamate--ammonia ligase | 2.57 | | 0.30 | 2.72 | 0.17 | | Amino acid biosynthesis | no |  | no |  |
| 16331651 | cyanate hydratase | 1.52 | | 0.32 | 1.50 | 0.25 | | Amino acid biosynthesis | no |  | no |  |
| 16332126 | ketol-acid reductoisomerase | 1.52 | | 0.21 | 1.57 | 0.18 | | Amino acid biosynthesis | no |  | no |  |
| 16331650 | ferredoxin--nitrite reductase | 1.86 | | 0.18 | 1.69 | 0.28 | | Amino acid metabolism | [19] | long term only | [10] | short term only |
| 16330763 | UDP-N-acetylmuramoylalanyl-D-glutamyl-2,6-diamino-pimelate-D-alanyl-D-alanine ligase | 1.61 | | 0.29 | 1.64 | 0.27 | | Cell envelope | no |  | no |  |
| 16332281 | heat shock protein 90 | 2.24 | | 0.22 | 1.90 | 0.20 | | Cellular processes | no |  | both | short term only |
| 16330619 | superoxide dismutase | 2.83 | | 0.13 | 2.83 | 0.26 | | Cellular processes | [19] | short and long term | [18] | short term only |
| 16331261 | molecular chaperone DnaK | 1.59 | | 0.20 | 1.83 | 0.20 | | Cellular processes | [19] | short and long term | [18] |  |
| 16329588 | 16.6 kDa small heat shock protein | 1.75 | | 0.17 | 2.10 | 0.35 | | Cellular processes | [19] | short term only | [10] | short term only |
| 16330002 | co-chaperonin GroES | 2.61 | | 0.28 | 2.93 | 0.35 | | Cellular processes | [19] | short term only | [10] | short term only |
| 16329392 | 4-alpha-glucanotransferase | 2.78 | | 0.00 | 3.00 | 0.00 | | Central intermediary metabolism | [19] | long term only | no |  |
| 16330944 | Glucosyl-glycerol-phosphate synthase | 4.86 | | 0.00 | 6.21 | 0.13 | | Central intermediary metabolism | no |  | no |  |
| 16332282 | glucose-1-phosphate adenylyltransferase | 1.62 | | 0.30 | 1.66 | 0.25 | | Central intermediary metabolism | no |  | no |  |
| 16331386 | fructose-bisphosphate aldolase | 1.52 | | 0.27 | 1.55 | 0.27 | | Energy metabolism | no |  | [10] | short term only |
| 16331115 | phosphoglycerate kinase | 2.68 | | 0.26 | 2.99 | 0.24 | | Energy metabolism | [19] | Long term | no |  |
| 16331208 | dihydrolipoamide acetyltransferase | 2.18 | | 0.31 | 2.23 | 0.14 | | Energy metabolism | no |  | no |  |
| 16330943 | glycerol-3-phosphate dehydrogenase | 1.95 | | 0.00 | 1.52 | 0.00 | | Fatty acid, phospholipid and sterol metabolism | no |  | [10] | short and long term |
| 16331180 | hypothetical protein sll1863 | 10.72 | | 0.25 | 4.74 | 0.33 | | Hypothetical | [19] | short and long term | [10] | short and long term |
| 16331737 | hypothetical protein slr0846 | 1.50 | | 0.34 | 1.70 | 0.24 | | Hypothetical | no |  | [18] |  |
| 16329942 | hypothetical protein slr1894 | 1.99 | | 0.19 | 1.90 | 0.17 | | Hypothetical | [19] | short and long term | [10] | short and long term |
| 16330945 | hypothetical protein slr1670 | 4.17 | | 0.27 | 5.38 | 0.40 | | Hypothetical | no |  | [10] | short and long term |
| 16329773 | hypothetical protein slr1039 | 2.14 | | 0.00 | 2.67 | 0.00 | | Hypothetical | no |  | no |  |
| 16330217 | hypothetical protein sll0861 | 3.57 | | 0.01 | 4.53 | 0.05 | | Hypothetical | no |  | no |  |
| 16330542 | hypothetical protein sll0245 | 1.75 | | 0.36 | 1.85 | 0.38 | | Hypothetical | no |  | no |  |
| 16330922 | hypothetical protein slr1161 | 1.69 | | 0.13 | 1.70 | 0.17 | | Hypothetical | no |  | no |  |
| 16331360 | hypothetical protein sll0319 | 2.41 | | 0.24 | 2.55 | 0.24 | | Hypothetical | no |  | no |  |
| 16332006 | hypothetical protein slr0042 | 1.96 | | 0.39 | 1.98 | 0.32 | | Hypothetical | no |  | no |  |
| 16332072 | hypothetical protein slr0551 | 2.29 | | 0.36 | 1.74 | 0.36 | | Hypothetical | no |  | no |  |
| 16330514 | circadian clock protein KaiC | 3.23 | | 0.41 | 3.58 | 0.44 | | Other categories | no |  | [10] | long term |
| 16332149 | aconitate hydratase | 1.65 | | 0.06 | 1.66 | 0.13 | | Other categories | no |  | no |  |
| 131288 | photosystem II 44 kDa reaction center protein | 1.82 | | 0.74 | 1.80 | 0.32 | | Photosynthesis and respiration | [33] |  | no |  |
| 16330219 | apocytochrome f precursor | 1.93 | | 0.19 | 1.69 | 0.07 | | Photosynthesis and respiration | no |  | no |  |
| 16331216 | light repressed protein | 2.62 | | 0.20 | 2.52 | 0.25 | | Regulatory proteins | [33] |  | no |  |
| 16329957 | DNA-directed RNA polymerase beta subunit | 1.60 | | 0.35 | 1.70 | 0.32 | | Transcription | no |  | no |  |
| 16329913 | 30S ribosomal protein S9 | 2.01 | | 0.20 | 1.81 | 0.21 | | Translation | [19] | short term only | [18] | short term only |
| 16329925 | 30S ribosomal protein S5 | 1.74 | | 0.20 | 1.70 | 0.17 | | Translation | [19] | short term only | [18] | short term only |
| 16329927 | 50S ribosomal protein L6 | 1.54 | | 0.35 | 1.67 | 0.27 | | Translation | [19] | short term only | [18] | short term only |
| 16329935 | 30S ribosomal protein S3 | 1.68 | | 0.25 | 1.71 | 0.25 | | Translation | [19] | short term only | [18] | short term only |
| 16329941 | 50S ribosomal protein L3 | 2.99 | | 0.84 | 2.27 | 0.34 | | Translation | [19] | short term only | [18] | short term only |
| 16330008 | 50S ribosomal protein L7/L12 | 2.46 | | 0.19 | 1.88 | 0.23 | | Translation | [19] | short term only | [18] | short term only |
| 16329482 | translation initiation factor IF-2 | 2.38 | | 0.26 | 2.09 | 0.09 | | Translation | no |  | [10] | short term only |
| 16329288 | translation initiation factor IF-2 | 1.61 | | 0.05 | 1.66 | 0.15 | | Translation | no |  | [10] | short term only |
| 16330189 | RNA-binding protein | 1.59 | | 0.39 | 1.75 | 0.27 | | Translation | [19] | short term only | [10] | short term only |
| 16332012 | RNA-binding protein | 2.21 | | 0.11 | 2.22 | 0.00 | | Translation | [19] | short term only | [10] | short term only |
| 16329356 | elongation factor EF-G | 2.91 | | 0.21 | 3.17 | 0.38 | | Translation | no |  | no |  |
| 16330433 | peptidyl-prolyl cis-trans isomerase | 2.07 | | 0.17 | 2.40 | 0.18 | | Translation | no |  | no |  |
| 16331631 | glutamyl-tRNA synthetase | 1.83 | | 0.15 | 2.32 | 0.08 | | Translation | [19] | long term only | no |  |
| 16332123 | elongation factor EF-2 | 1.70 | | 0.17 | 1.79 | 0.16 | | Translation | no |  | no |  |
| 16329924 | 50S ribosomal protein L15 | 2.58 | | n/a | 1.95 | 0.36 | | Translation | no |  | no |  |
| 16331324 | 50S ribosomal protein L35 | 2.29 | | 0.28 | 2.63 | 0.32 | | Translation | no |  | no |  |
| 16330084 | nitrate transport 45kD protein | 1.83 | | 0.14 | 1.52 | 0.20 | | Transport and binding | [33] | long term only | [18] | short term only |
| 16331408 | ABC1-like | 4.27 | | 0.11 | 4.93 | 0.16 | | Transport and binding | no |  | no |  |
| 16331744 | ABC transporter subunit | 2.35 | | 0.41 | 2.66 | 0.30 | | Transport and binding | no |  | no |  |
| **DECREASED EXPRESSION IN 6% ACCLIMATED CELLS** | | | | | | | | | | | | |
| 16330243 | anti-sigma B factor antagonist | 2.70 | 0.51 | | 2.30 | 0.22 | | Unknown | no |  | no |  |
| 16330576 | acetolactate synthase | 1.70 | 0.06 | | 1.75 | 0.27 | | Amino acid biosynthesis | no |  | no |  |
| 16331281 | 2-isopropylmalate synthase | 2.09 | 0.14 | | 2.40 | 0.73 | | Amino acid biosynthesis | no |  | no |  |
| 16330473 | glutathione peroxidase | 1.74 | 0.28 | | 1.99 | 0.24 | | Biosynthesis of cofactors, prosthetic groups, and carriers | [19] | opposite | no |  |
| 3023789 | Probable dihydroneopterin aldolase (DHNA) | 1.74 | 0.31 | | 1.85 | 0.36 | | Biosynthesis of cofactors, prosthetic groups, and carriers | no |  | no |  |
| 16331030 | molybdopterin biosynthesis protein MoeB | 1.64 | 0.48 | | 1.58 | 0.26 | | Biosynthesis of cofactors, prosthetic groups, and carriers | [33] | opposite | no |  |
| 16331608 | GTP cyclohydrolase I | 2.61 | 0.11 | | 3.54 | 0.24 | | Biosynthesis of cofactors, prosthetic groups, and carriers | no |  | no |  |
| 16331297 | cell division cycle protein | 1.62 | 0.27 | | 1.77 | 0.36 | | Cell division | no |  | no |  |
| 16330088 | cell division protein FtsZ | 1.72 | 0.21 | | 1.61 | 0.21 | | Cellular processes | no |  | [18] | short term increase |
| 16331493 | heat shock protein; GrpE | 1.54 | 0.21 | | 1.87 | 0.25 | | Cellular processes | no |  | no |  |
| 16329675 | urease alpha subunit | 1.64 | 0.23 | | 1.90 | 0.12 | | Central internediary metabolism | no |  | no |  |
| 16330143 | glycogen phosphorylase | 2.68 | 0.21 | | 1.83 | 0.04 | | Central internediary metabolism | [19] |  | no |  |
| 16330360 | OxPPCycle gene | 1.52 | 0.44 | | 1.70 | 0.26 | | Central internediary metabolism | no |  | no |  |
| 16331023 | inorganic pyrophosphatase | 2.56 | 0.27 | | 2.23 | 0.15 | | Central internediary metabolism | no |  | no |  |
| 16331412 | bifunctional GMP synthase/glutamine amidotransferase | 1.76 | 0.26 | | 1.99 | 0.24 | | Central internediary metabolism | no |  | no |  |
| 16331862 | polyphosphate kinase | 1.70 | 0.35 | | 2.20 | 0.76 | | central internediary metabolism | no |  | no |  |
| 16331645 | hypothetical protein sll0887 | 2.05 | n/a | | 1.56 | 0.22 | | DNA replication, restriction, modification, recombination, and repair | no |  | no |  |
| 2506772 | Isocitrate dehydrogenase [NADP] | 1.70 | 0.08 | | 1.61 | 0.18 | | Energy metabolism | no |  | no |  |
| 16329656 | glutamate decarboxylase | 2.79 | n/a | | 1.69 | 0.18 | | Energy metabolism | no |  | no |  |
| 16330043 | glucose-6-phosphate 1-dehydrogenase | 3.74 | 0.59 | | 2.33 | 0.42 | | Energy metabolism | no |  | no |  |
| 16331307 | 6-phosphogluconate dehydrogenase | 2.12 | 0.14 | | 1.90 | 0.12 | | Energy metabolism | no |  | no |  |
| 16331394 | ribulose bisphosphate carboxylase small SU | 1.90 | 0.19 | | 2.15 | 0.19 | | Energy metabolism | no |  | no |  |
| 16331464 | citrate synthase | 1.58 | 0.15 | | 1.57 | 0.12 | | Energy metabolism | no |  | no |  |
| 16329546 | hypothetical protein slr0670 | 1.52 | 0.48 | | 2.36 | 0.24 | | Hypothetical | no |  | no |  |
| 16329841 | hypothetical protein slr2005 | 3.84 | 0.03 | | 2.33 | 0.03 | | Hypothetical | no |  | no |  |
| 16330234 | hypothetical protein sll1305 | 2.56 | 0.26 | | 2.20 | 0.23 | | Hypothetical | no |  | [18] |  |
| 16330239 | hypothetical protein slr1852 | 3.55 | 0.19 | | 3.59 | 0.08 | | Hypothetical | no |  | [18] |  |
| 16330242 | hypothetical protein slr1855 | 4.18 | 0.19 | | 4.68 | 0.15 | | Hypothetical | no |  | [18] |  |
| 16330319 | hypothetical protein slr2144 | 3.42 | 0.29 | | 2.57 | 0.03 | | Hypothetical | [19] |  | no |  |
| 16330744 | hypothetical protein slr1338 | 1.97 | 0.23 | | 1.90 | 0.11 | | Hypothetical | no |  | no |  |
| 16330813 | hypothetical protein sll1873 | 1.89 | 0.26 | | 1.90 | 0.31 | | Hypothetical | no |  | no |  |
| 16331085 | hypothetical protein sll0272 | 1.98 | 0.08 | | 3.22 | 0.19 | | Hypothetical | no |  | no |  |
| 16331120 | hypothetical protein sll0359 | 2.21 | 0.16 | | 2.09 | 0.15 | | Hypothetical | no |  | no |  |
| 16332260 | hypothetical protein sll0446 | 1.55 | 0.42 | | 1.66 | 0.03 | | Hypothetical | no |  | no |  |
| 16332268 | putative phosphoketolase | 3.76 | 0.17 | | 3.75 | 0.16 | | Hypothetical | no |  | no |  |
| 16332299 | hypothetical protein sll1106 | 1.57 | 0.17 | | 1.51 | 0.15 | | Hypothetical | no |  | [10] | opposite |
| 38505793 | hypothetical protein sll8018 | 1.62 | 0.11 | | 1.65 | 0.02 | | Hypothetical | no |  | no |  |
| 2499211 | protein drgA | 2.32 | 0.06 | | 2.51 | 0.18 | | Other categories | no |  | no |  |
| 16329322 | potential FMN-protein | 2.03 | 0.57 | | 2.06 | 0.27 | | Other categories | no |  | no |  |
| 16329908 | short chain dehydrogenase | 2.28 | 0.07 | | 2.23 | 0.08 | | Other categories | no |  | no |  |
| 16329971 | rehydrin | 1.60 | 0.22 | | 1.57 | 0.18 | | Other categories | no |  | no |  |
| 16330683 | hydrogenase large subunit | 1.62 | 0.19 | | 1.57 | 0.14 | | Other categories | no |  | no |  |
| 16330967 | soluble hydrogenase 42 kD subunit | 1.83 | 0.05 | | 1.58 | 0.12 | | Other categories | no |  | no |  |
| 16332300 | zinc-containing alcohol dehydrogenase family | 1.87 | 0.18 | | 2.23 | 0.35 | | Other categories | no |  | no |  |
| 16329366 | carbon dioxide concentrating mechanism | 2.25 | 0.16 | | 2.63 | 0.36 | | Photosynthesis and respiration | no |  | no |  |
| 16329478 | allophycocyanin-B | 1.81 | 0.21 | | 2.54 | 0.10 | | Photosynthesis and respiration | no |  | [18] |  |
| 16329710 | phycobilisome rod-core linker polypeptide | 1.85 | 0.29 | | 1.86 | 0.24 | | Photosynthesis and respiration | no |  | [18] |  |
| 16329820 | phycocyanin associated linker protein | 3.86 | 0.23 | | 4.66 | 0.17 | | Photosynthesis and respiration | no |  | [18] |  |
| 16329821 | phycocyanin associated linker protein | 3.88 | 0.34 | | 4.25 | 0.29 | | Photosynthesis and respiration | no |  | [18] |  |
| 16329822 | phycocyanin associated linker protein | 3.79 | 0.35 | | 4.80 | 0.41 | | Photosynthesis and respiration | no |  | [18] |  |
| 16329823 | phycocyanin a subunit | 3.13 | 0.21 | | 4.32 | 0.28 | | Photosynthesis and respiration | no |  | no |  |
| 16329824 | phycocyanin b subunit | 2.19 | 0.29 | | 2.51 | 0.25 | | Photosynthesis and respiration | no |  | no |  |
| 16330467 | allophycocyanin b | 1.56 | 0.22 | | 1.68 | 0.20 | | Photosynthesis and respiration | no |  | [18] |  |
| 16330539 | flavodoxin | 1.83 | 0.06 | | 2.06 | 0.19 | | Photosynthesis and respiration | [33] | opposite | no |  |
| 16331106 | cytochrome c550 | 1.93 | 0.23 | | 2.16 | 0.18 | | Photosynthesis and respiration | no |  | no |  |
| 16331547 | chloroplast membrane-assoc 30 kD protein | 1.66 | n/a | | 1.63 | n/a | | Photosynthesis and respiration | no |  | no |  |
| 16332085 | NADH dehydrogenase subunit I | 1.59 | 0.16 | | 1.75 | 0.08 | | Photosynthesis and respiration | no |  | no |  |
| 16329790 | CheA like protein | 3.40 | n/a | | 2.23 | 0.31 | | Regulatory | no |  | no |  |
| 16330362 | SOS function regulatory protein | 1.66 | 0.26 | | 1.61 | 0.34 | | Regulatory | no |  | no |  |
| 16331356 | transcriptional regulatory protein | 1.72 | n/a | | 1.73 | n/a | | Regulatory | no |  | no |  |
| 16331742 | sensory transduction histidine kinase | 2.00 | 0.30 | | 2.96 | 0.38 | | Regulatory | no |  | no |  |
| 16329303 | peptidyl-prolyl cis-trans isomerase B | 1.85 | 0.05 | | 2.04 | 0.09 | | Translation | no |  | no |  |
| 16329479 | S-adenosylmethionine synthetase | 1.63 | 0.09 | | 1.52 | 0.37 | | Translation | no |  | no |  |
| 16329894 | leucyl-tRNA synthetase | 1.59 | 0.15 | | 2.01 | 0.11 | | Translation | no |  | no |  |
| 16329909 | 50S ribosomal protein L25 | 2.00 | 0.20 | | 1.83 | 0.14 | | Translation | no |  | no |  |
| 16330739 | 30S ribosomal protein S2 | 2.09 | n/a | | 3.02 | n/a | | Translation | no |  | no |  |
| 16331151 | ribosome releasing factor | 1.55 | 0.07 | | 1.99 | 0.11 | | Translation | no |  | no |  |
| 16331161 | ATP-dependent Clp protease proteolytic SU | 2.38 | 0.37 | | 2.29 | 0.27 | | Translation | no |  | no |  |
| 16331163 | aminopeptidase P | 2.28 | 0.15 | | 1.81 | 0.57 | | Translation | no |  | no |  |
| 16331761 | threonyl-tRNA synthetase | 1.95 | 0.09 | | 1.79 | 0.15 | | Translation | no |  | no |  |
| 16329434 | iron transport protein | 1.75 | 0.19 | | 1.99 | 0.23 | | Transport and binding | no |  | no |  |
| 16330429 | periplasmic phosphate binding protein | 10.07 | 0.21 | | 10.38 | 0.17 | | Transport and binding | [33] | opposite | no |  |
| 16330540 | iron-stress chlorophyll-binding protein | 20.65 | 0.34 | | 29.62 | 0.20 | | Transport and binding | no |  | no |  |
| 16331793 | periplasmic iron-binding protein | 9.28 | 0.56 | | 11.08 | 0.54 | | Transport and binding | [33] | opposite | no |  |
| 16332004 | bicarbonate transporter | 2.09 | 0.36 | | 3.62 | 0.24 | | Transport and binding | [33] | opposite | [19] | opposite |
| 16332097 | bacterioferritin | 1.61 | 0.17 | | 1.72 | 0.14 | | Transport and binding proteins | no |  | no |  |
| 16330022 | poly(3-hydroxyalkanoate) synthase | 2.81 | n/a | | 3.47 | n/a | | Unknown | no |  | no |  |

: Functional groups designated by Kazusa (http://bacteria.kazusa.or.jp/cyanobase/Synechocystis/)

* Studies in which protein or transcript levels have previously been identified as differentially expressed (*opposite* labelled proteins refer to proteins where the direction of expression is opposite (i.e. reduced instead of increased expression and vice versa).
