# Supplementary material for: A systems biology approach to investigate the response of Synechocystis sp. PCC6803 to a high salt environment
Source: Saline Syst. 2009 Sep 7;5:8. doi: 10.1186/1746-1448-5-8 (PMC2743698; doi:10.1186/1746-1448-5-8)
Supplement: Additional file 4 — Table S3. Primer (oligonucleotide) sequences designed for each protein-coding gene (Ta = annealing temperature). [file 1746-1448-5-8-S4.doc]

Table S3: Primer (oligonucleotide) sequences designed for each protein-coding gene (T_a_ = annealing temperature).

| gI accession | Protein name | Gene name | Forward sequence | No. of bases | Reverse sequence | No. of bases | T_a_ | Amplicon length |
| --- | --- | --- | --- | --- | --- | --- | --- | --- |
| 16331408 | ABC1-like | *UbiB* | TCGCCGGTTTTCAACTTACC | 20 | TATCTCCGGAGCCCATTGC | 19 | 61 | 67 |
| 16330243 | Anti-sigma B factor antagonist | *IcfG* | CGCCAACTCAGCCCAATTA | 19 | CACGGCCATGGTGTCGAT | 18 | 60 | 64 |
| 16331161 | ATP-dependent Clp protease proteolytic subunit | *ClpP* | ACTTGGGAATGCCGCTTTT | 19 | TCAATGCCGACCTGTTGCT | 19 | 60 | 55 |
| 16332004 | Bicarbonate transporter | *CmpA* | GTGCCTCCCGCTGAAACA | 18 | TCTCCAGTGCTAAAAGCATCCA | 22 | 61 | 65 |
| 16330088 | Cell division protein FtsZ | *FtsZ* | AAATGGACGGGTGACAATGC | 20 | CCGAGGTGGCCAAAGAAAT | 19 | 60 | 56 |
| 16330514 | Circadian clock protein KaiC | *KaiC* | GGCCGCCAGGGTAGTTTT | 18 | CGTGGCAGTAGCATTTTAGTAACC | 24 | 61 | 60 |
| 16330002 | Co-chaperonin GroES | *GroES* | CCTACTCCCCTGTGGAAGTCAA | 22 | GTGCCGGCATACTTGGAATAG | 21 | 59 | 61 |
| 16330473 | Glutathione peroxidase | *GshP* | AGGTGGTGGCTCGCTTTG | 18 | GCCGCCTTGAGATTAGTGTCA | 21 | 60 | 58 |
| 16332299 | Hypothetical protein sll1106 | sll1106 | CACAACGGCGGCATCTTC | 18 | GCCCTGGCCAAGCTACAA | 18 | 59 | 57 |
| 16331261 | Molecular chaperone DnaK | *DnaK* | ATCCCGAATGGCATTTTCC | 19 | CCGGGCCAAGTTTGAAGAA | 19 | 58 | 76 |
| 16330429 | Periplasmic phosphate binding protein | *PstS* | TTGGTGCCACCGATGCT | 17 | CCACACCCCGTTTCATCTGT | 20 | 61 | 60 |
| 16331151 | Ribosome releasing factor | *Frr* | CGCATCCCGGCGAATAT | 17 | GCAGGTAAATTGGCGGAAGA | 20 | 60 | 57 |
| 16330362 | SOS function regulatory protein | *LexA* | GGACGCAGAGGAAGTGGAA | 19 | ATGACCCTGAGGGCAAAAAC | 20 | 58 | 75 |
| 16330619 | Superoxide dismutase | *Sod* | AGCACCCTGGAGTTCCATCA | 20 | AGTACCCGCCACTGCATTGT | 20 | 59 | 75 |
